# Rethinking workforce planning for integrated care: using scenario analysis to facilitate policy development

**DOI:** 10.1186/s12913-020-05304-4

**Published:** 2020-05-15

**Authors:** Gareth H. Rees, Peter Crampton, Robin Gauld, Stephen MacDonell

**Affiliations:** 1 ESAN University, Alonso de Molina 1652, Monterrico Chico, Lima 33, Peru; 2 Otago Medical School and Centre for Health Systems and Technology, University of Otago, PO Box 56, Dunedin, 9054 New Zealand; 3 Dean’s Office, Otago Business School and Centre for Health Systems and Technology, University of Otago, PO Box 56, Dunedin, 9054 New Zealand; 4 Department of Information Science and Centre for Health Systems and Technology, Otago Business School, University of Otago, PO Box 56, Dunedin, 9054 New Zealand

**Keywords:** Integrated care, Health workforce planning, Health policy, Health workforce governance, Scenario analysis, Mixed methods, New Zealand

## Abstract

**Background:**

A goal of health workforce planning is to have the most appropriate workforce available to meet prevailing needs. However, this is a difficult task when considering integrated care, as future workforces may require different numbers, roles and skill mixes than those at present. With this uncertainty and large variations in what constitutes integrated care, current health workforce policy and planning processes are poorly placed to respond. In order to address this issue, we present a scenario-based workforce planning approach.

**Methods:**

We propose a novel mixed methods design, incorporating content analysis, scenario methods and scenario analysis through the use of a policy Delphi. The design prescribes that data be gathered from workforce documents and studies that are used to develop scenarios, which are then assessed by a panel of suitably qualified people. Assessment consists of evaluating scenario desirability, feasibility and validity and includes a process for indicating policy development opportunities.

**Results:**

We confirmed our method using data from New Zealand’s Older Persons Health sector and its workforce. Three scenarios resulted, one that reflects a normative direction and two alternatives that reflect key sector workforce drivers and trends. One of these, based on alternative assumptions, was found to be more desirable by the policy Delphi panel. The panel also found a number of favourable policy proposals.

**Conclusions:**

The method shows that through applying techniques that have been developed to accommodate uncertainty, health workforce planning can benefit when confronting issues associated with integrated care. The method contributes to overcoming significant weaknesses of present health workforce planning approaches by identifying a wider range of plausible futures and thematic kernels for policy development. The use of scenarios provides a means to contemplate future situations and provides opportunities for policy rehearsal and reflection.

## Background

Health workforce planning seeks to ensure the right people receive the right services at the right place, at the right time, from those with the right skills [1] and at the right price or cost [2]. This task has become increasingly challenging for workforce planners as health care embraces increasing integration [3], as a future health workforces’ configurations may not be known [4], may be quite different from those at present [5] and may require people to possess different or strengthened competencies [3]. These uncertainties are exacerbated by the current medical education settings, which do not cater well for the teaching of integrated care practices [6, 7].

Adding to this is the problem that integrated care is ill defined, though it is broadly considered to be an inter-sectoral approach that aims to align the health care system with other human service systems [8]. There is an array of definitions of integration and how integrated care is configured, implemented and coordinated, however these have the common aims of improving outcomes for the target population, enhancing their quality of life and improving consumer satisfaction [9]. Moreover, as a developing field, integrated care lacks policy clarity and has few of the hallmarks of systematic policy development and implementation [10]. Thus, the understanding of integrated care and its implementation may require the use of other than traditional research approaches to provide needed insights [11]. In response, we propose a contrasting approach to traditional health workforce planning. Rather than attempting to predict future integrated care workforce roles or numbers, we present a novel method as a means to contribute to a better understanding of how integrated care workforces may evolve and to reveal associated policies.

Thus, the aim of the article is to present and discuss our method. We continue by outlining the planning context, the characteristics of integrated care that act to limit traditional workforce planning approaches, and the narrow use of scenarios in current workforce planning. Next, we detail our mixed methods approach, which acts to reduce uncertainty and to provide improved clarity of workforce structures and policy components. Finally, we outline the results of an application of the method and discuss its utility.

### Integrated care in New Zealand

A number of countries have developed strategies or policies intended to promote the integration of care [12, 13]. New Zealand, as one of these, has integrated some of its health planning and funding functions, but has experienced few service improvements [14]. The nation’s integrated care focus is enabled by its Health Strategy, which identifies an “integrated approach” as key to outcomes [15], p.1, although more effective ways of working and planning have been advised [16].

**Fig. 1:**
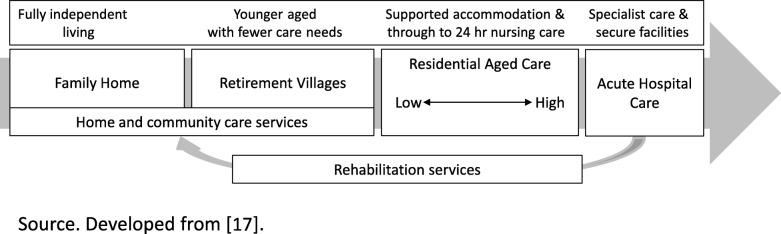
New Zealand’s Older Persons Health Service Care Continua

A sector that can benefit from such improved service and workforce planning is that of Older Persons Health (OPH), a sub-sector that serves a growing population [17] with multiple health and social care needs requiring multidisciplinary collaboration and coordination [18]. New Zealand’s rates of people over 65 being in care have steadily fallen since the 1970s to less than 7 % in the mid-2000s and is attributed to a wider acceptance of community living, changes to state residential care subsidy criteria and a broadening of community-based support services [19]. OPH in New Zealand ranges from fully independent living to specialist and secure care facilities [17] (see Fig. 1), with more than half of those in residential facilities being 85 years or older [19]. A result of the high numbers of older people residing outside of residential care has increased both the importance of the family’s role and the numbers of community based support and care workers [17]. These workers are predominantly female, older, part-time and lowly paid [20].

### Integrated care and health workforce planning

Integrated care has been described as a conceptual “imprecise hodgepodge”, with diverse meanings and multiple interactions between numerous actors, hampering its systematic understanding and successful real-world application [21], p.12. As such, integration can be orientated: for individuals, as case management or care plans; for groups, as models for chronic care or the frail and elderly; for specific long-term conditions such as diabetes or cardiovascular disease; or for populations [22]. This diversity has policy quality effects, impacts on implementation quality [23] and adds to stakeholder confusion over integration’s purpose or how to proceed [11], particularly when inter-professional collaboration is dictated by integrated service designs [24].

Thus, integrated care and its lack of definitional regularity provides problems for traditional health workforce planning approaches. Mostly, these approaches are based on the matching of worker volumes with expected demand using highly simplistic models that are usually developed from historical allocations of single professions [2]. These models also tend to pay little attention to a population’s health needs [1] and the variations that may exist within [25], thereby producing poor results [2] and perpetuating the health system status quo [26]. Though, while these limitations are well known, few health workforce planning systems are able to cater for the requirements of integrated care [27]. While there are a few approaches that enable planners to review, revise and remodel roles, tasks and worker numbers when designing such services [25, 28, 29], these methods may not be practical over longer time horizons or when it is difficult to quantify role-mix or service innovation impacts [26].

When faced with uncertainty, such as that posed by integrated care, scenarios are an appropriate method to gain situational understanding or to explore a particular issue [30]. As such they can be used to improve organisational integration, communication and learning [31], to examine conditions of uncertainty, to understand intractable problems [32, 33], and to formulate strategic responses and decisions [34]. Van der Heijden [34] notes that scenarios aid decision making in two ways, through interactive rational analysis or through identifying and describing the social interactions and processes, which lead to a future.

Many health workforce planning scenarios reported in the literature tend to be quantitative in nature [35], applying what-if or predictive scenarios as a means to scout planning dimensions or estimating what is expected to occur in the short term [36]. However another, the qualitative scenario, is also capable of assisting health workforce planning [37]. This approach presents scenarios as descriptive narratives [38], which may: be normative, where the scenario has explicit, accepted or normative starting points and focuses on how certain future situations or objectives can be realised or what we should do; or, be exploratory, which aim to explore a variety situations or developments that are regarded as possible [36]. While the qualitative scenario features less in the health workforce literature [39], their use can provide policy makers with perspectives that are “devoid of current constraints, vested interests and current concerns” and with space to consider the important over the urgent or to “focus on what really matters” ([40], p.4).

The reluctance to use such descriptive scenarios in public policy making has been attributed to scenarios’: broadness, making them difficult for particular policy development [41]; timescales, which are generally longer than those of the policy maker [42]; difficulty of merging scenarios with the policy making process [43]; and their easy dismissal on grounds of credibility, legitimacy, and salience [44]. Moreover, public institutions tend to be intolerant of uncertainty, driven by a belief that predicting the future is reliable [45]. These institutions’ policy makers tend to have an affinity for and a reliance on numerical data, even if its accuracy is questionable [46] and face political pressure to avoid mistakes [47]. The reluctance is despite the knowledge that to strengthen policy analysis we must explore its methodological challenges [48] and apply a wider range of methods [49].

Considering these issues, we developed an approach that allows us to better address workforce planning uncertainties, such as those posed by integrated care, and to uncover policy development possibilities. Before implementing our approach, we applied for and received ethical approval from our host university.

## Methods

### Approach overview

Mixed Methods (MM) is an increasingly common health care research approach that intentionally integrates a number of methods [50], which provides a reliable and valid way to build on one method’s findings of by applying another, integrating types of data, or embedding one analysis within another [51]. These actions require more involved reporting, with researchers recommended to describe, justify and present their designs, methods and results clearly and transparently to reveal their study’s unique insights [52–54].

**Fig. 2:**
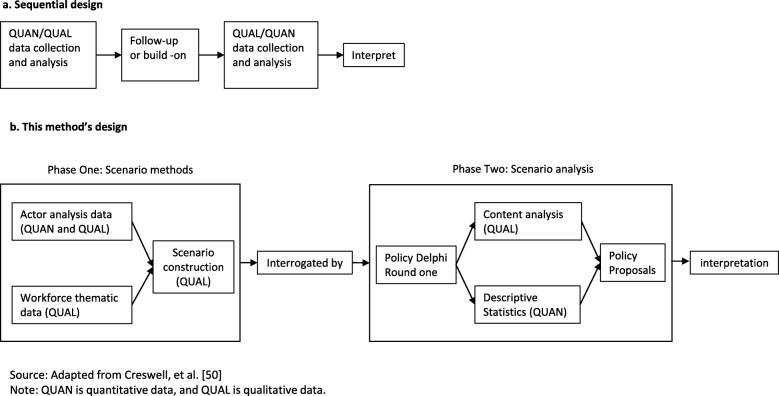
The method's design

Our design, presented in Fig. 2, is sequential [50], where we firstly construct the scenarios by applying scenario methods [31] with recent workforce thematic and actor (stakeholder) data, followed by scenario analysis, which is the application or use of scenarios by individuals or groups in order to explore a particular issue [30]. For the analysis, we used the structured group communication technique, the policy Delphi, which is appropriate in this context as it seeks to generate the strongest possible opposing views for a policy issue [55] by forcing participants to think about issue pros and cons [56].

### Scenario methods

There is no single way to construct scenarios [57], though Glenn and The Futures Group International [38] describe eight approaches that share the common stages of diagnosis, development and reporting [58]. Taking note of these, we took our scenario construction data from two sources. The first being an examination of eight workforce documents that reflect a future state of OPH service delivery and workforce issues (see Table 1). These documents were identified as part of a wider structured grey literature review, which applied targeted searches within government, multinational agencies and national health research organisation websites.

**Table 1:** Workforce documents used to construct the OPH normative scenario

Document type	OPH specific	General workforce
Work Service Review^a^	Workforce for the Care of Older People	Maori Health Workforce Priorities Pacific Health Workforce Service Forecast Palliative Care Workforce Service Review Rehabilitation Service and Workforce Forecast Diabetes Work Service Review
Workforce projection model		Rural Nursing Workforce
Workforce report (new role)		Nurse Practitioners in New Zealand Registered Nurse Prescribing in Diabetes Care

*Source.* Documents accessed through http://www.health.govt.nz/our-work/health-workforce/workforce- service-forecasts and http://www.health.govt.nz/our-work/health-workforce/new-roles-and- initiatives/established-initiatives

^a^ Developed by HWNZ, a Work Service Review (WSR) is a service-aggregated, clinician-led and patient-centred scenario that identifies future possible model(s) of care [59]

Using the Google site search function (where the term “site:websiteURL” is inserted after the search terms), which limits the search solely to the website selected, we searched New Zealand’s Ministry of Health website. We used the search terms ‘health workforce’, ‘forecasting’ and ‘health labour (labor) market’ and followed this by a full catalogue scan to ascertain that all possible documents had been identified. The selection identified those documents that directly concerned the OPH workforce or which mentioned older people as having conditions, being treated by and/or receiving care from one or more relevant workforces.

Our second source of data was taken from Rees et al. [60], which provided sufficient data and variations to construct a range of exploratory scenarios [41]. Taking the OPH data from this actor analysis of two New Zealand’s health system sub-sectors, provided us with the identification of critical factors for the future OPH workforce, the relative power of actors within the system and the divisiveness of identified issues. These data indicated which actors hold more power and the potential drivers of change for a future workforce system. As such, these are similar to data produced by a horizon scan, as these identify workforce trends and their implications that the actors saw as critical to the workforce’s future.

### Content analysis

We deductively content analysed the eight documents to identify and organised thematic codes [61]. We used a simple form of computer-assisted content analysis, code and retrieve [62] to extract the data, by applying the search terms ‘vision’, ‘model of care’ and ‘scenario’ as these were expected to provide suitable descriptions of the service’s vision, health needs, configurations, desired models of care and clinical scenarios to provide the objectives and intentions from which to construct a normative scenario [63]. From this process, we derived a range of data in terms of the workforce participants, their cultural, clinical and geographical contexts and service expectations, which together represent the normative or expected trajectory of the sector and indicate how the workforce should be constituted.

### Scenario development

We approached the scenario drafting by carefully following step-by-step procedures to plot the narratives using the headline method [57]: a process where headlines are expanded into a narrative utilising the unique combination of conditions provided by the data’s contributing themes. Thus, we took the main themes from the document content analysis and using these as headlines, built a description of how New Zealand’s OPH normative future was likely to develop. By taking the actor analysis’ divisive issues and potential behaviours we identified the alternative scenario main themes [64] and blended these with the normative to produce two exploratory alternatives. For these we applied a “crisis and response” technique to draft plot lines ([57], p.72), ensuring distinct differences and responses to reflect the necessary diversity and ambiguity between them [65].

Once each core narrative was complete, we identified signposts, such as projects or studies that elucidated the scenario’s trajectory (−e.g. the use of specialist nursing or more detailed care planning to improve care outcomes). Each scenario also contained open-ended questions which, with the signposts, are a means to assist readers to engage and connect the scenario to the everyday world [65]. We were careful when constructing the scenarios to keep them parsimonious while including sufficient data for the reader to be able to comprehend and evaluate the described situation. Once the scenarios were complete, we tested them with a small group of academics and practitioners to improve their believability and internal consistency [66].

### Scenario analysis

Here we employed the policy Delphi, a process where a group of anonymous participants are asked to provide a complete-as-possible picture of the issue under investigation through the group’s diversity and extreme views [67]. While infrequently applied in health compared to the conventional Delphi [56], the policy Delphi is useful for a context characterised by conflicting interests such as found in health workforce planning [68].

### Panel selection and recruitment

Who participates in a policy Delphi is central to ensuring high quality results [69]. Careful panel selection supports a policy Delphi’s reliability through ensuring appropriate levels of panellist knowledge, experience and understanding of the topic under study [55, 70–72]. As the policy Delphi’s aim is to promote varied opinions [55], a panel’s diversity is just as important [73]. We, therefore, used purposive sampling as our selection method [74], selecting the panellists “for the important information they can provide” ([75], p.236) and their “valuable ideas” [76], p.7. To capture a range of perspectives our sampling strategy followed Okoli and Pawlowski’s [77] advice by identifying potential panellists from contributors to New Zealand health workforce documents, conference proceedings and New Zealand tertiary education institution websites, who we assessed against the eligibility criteria listed in Table 2.

**Table 2:** Panellist eligibility criteria

Area	Criterion
Professional Experience	In excess of 10 years related to the sub-sector
	Progressive work history
Level of responsibility	Participate in strategic decision making or policy development
Range of positions	Organisation leader
	Medical or policy academic
	Senior clinician
	General practice leader or nurse specialist
	Consumer advocate
	Policy manager or advisor

A policy Delphi’s results depend on the dynamics of its processes rather than statistical power [77], so large panels may be not be advantageous [66]. Small Delphi panels are able to produce reliable results [78] when there is sufficient group heterogeneity and knowledge [79]. Consequently, there are no clear rules for panellist numbers [79], nor directions for setting panel sizes [78]. Lower limit panel suggestions range from five to ten, with upper limit suggestions between twenty and fifty [55, 77, 79, 80]. New Zealand’s small population size, health establishment and two medical schools limited the potential knowledgeable participant population from the start, meaning a smaller panel was more likely to result.

A major problem with Delphi panels is attrition. Sometimes referred to as response or participant fatigue, attrition has been attributed to poor instructions and workload guidance, impersonal communications, question volume and repetition [56, 81, 82]. With the likelihood of a small panel, we were sensitive to this issue, so we included attrition reduction measures within the panellists’ invitations, which included clear instructions, an estimate of workload [83] and the benefits and practical outcomes [84]. In acknowledgment of time-poor panellists, we also provided pre-constructed scenarios [85] and pre-tested items [56] as an effort to reduce the panellists’ engagement time. Never-the-less, Turoff [55] notes that some panellists will give up regardless.

To recruit the panel, we issued nineteen invitations to a range of practitioners (8), educationalists (8) and policy organisation representatives (3). We judged this number to be sufficient to provide our minimum starting size of ten panellists. The invitation also included an option to refer another person, should the invitee not be able to participate. The invitations resulted in fifteen replies, four of whom declined, and one referral, who also declined, leading to a final panel size of eleven (see Table 3).

**Table 3:** Summary data of the policy Delphi panellists

#	Age	Gender	Organisation	Position	Working years and expertise	Qual (if given)
1		F	Education	Senior Research Fellow	+ 20 academic & policy	PhD
2		F	Education	Senior Lecturer	+ 20 practice & academic	PhD
3		F	Practice	CEO	+ 20 practice& management	
4		F	Education	Head of School	+ 20 practice & academic	PhD
5		F	Practice	CEO	+ 20 practice & management	
6		M	Policy	Researcher	+ 20 research & practice	
7		M	Education	Senior Research Fellow	+ 20 research & practice	
8		M	Education	Professor	+ 20 practice & academic	PhD
9		M	Practice	Medical Director	+ 20 practice & adviser	
10		F	Education	Course Co-ordinator	+ 15 practice & academic	
11		M	Practice	Medical Specialist	+ 15 practice	

### Panel administration

To conduct the survey, we chose the forecasting-expert-designed, free-of-charge online survey management web platform, Delphi Decision Aid [80, 86]. Online administration can have positive effects on time, organisation, engagement and data handling [71, 87]. Before proceeding, the panellists received comprehensive instructions on how to access the web-based questionnaires and to answer the questions.

The first round consisted of a two-part questionnaire. The first part of the questionnaire focussed on scenario evaluation. This contained four- or five-point closed questions taken from Turoff’s [55] scales to judge a policy issue or situation. These questions ask the panellists to grade the scenario’s desirability, from 1 (very undesirable) to 4 (very desirable), probability 1 (very improbable) to 5 (very probable) and confidence in the validity of the scenario’s premises from 1 (unreliable) to 4 (certain). The four-point rating scales contain no middle option as a means to prevent fence sitting, though a no-opinion option was also provided. The second part of the questionnaire required the panellists to answer open-ended engagement questions, to develop policy statements. To derive these statements, we applied an inductive content analysis to identify referential codes. By phrasing these codes as should-type statements, we developed parsimonious policy statements following Martino’s [70] recommended 25 word maximum and noting their relative importance by recording the code frequency per statement [88].

From the second round on, the panellists answered another two-part questionnaire, part one re-rating the scenarios and part two rating the policy statements. Policy statement rating was based on Turoff’s four-point scales for desirability and feasibility [55]. These dimensions’ ratings indicate a policy statement’s attractiveness and practicality and are intended to be a means for the respondents to rehearse or visualise the results of the statement’s implied actions.

As feedback is considered to be important to the Delphi process [89], panellists were provided with descriptive statistics and narrative response summaries following each round [79]. Panellists were also able to review the round responses online. As a question’s response stability was reached, it was eliminated from subsequent rounds progressively acting to reduce the questionnaire size and completion times.

A Delphi generally closes when stability of the panel’s responses is attained [90]. A simple method for measuring a policy Delphi response stability is Scheibe et al.’s [91] net percentage change approach, which calculates the net percentage change between each round’s data distribution and focusses on the group response as a whole, rather than the variation of individual responses. We chose this method for its ease of use and because as it accommodates non-normal or bi-modal data distributions that can be found in policy Delphi data. Due to the modest panel size, we set the response stability acceptability criterion at 20% as per Nelson [92], meaning that when the percentage change is less than 20% it is considered to be stable. It may take up to four or five rounds to achieve response stability, as panellist divisions tend to become more polarised in latter rounds [55].

We must also reveal here that the online website did not always faithfully collect or report the respondent data. Some items returned erroneous results and technical issues with the site were also encountered during questionnaire administration. When corrupt data were returned, the affected question was repeated. This acted to prevent some items reaching the stability threshold and thus provided a few indeterminate results.

## Results

### Results of content analysis

Using the document search terms, a number of critical concerns were found. These grouped into three thematic areas: (1) the workforce system as it is, (2) desired workforce outcomes and (3) particular workforce issues that are required to be addressed to realise these outcomes. A summary of the content analysis is presented in Table 4.

**Table 4:** Summary of content analysis

Theme	Critical concerns
Workforce system	Acute hospital care not adequate for presenting needs; Focus on episodic care; consequences from a reorientation of workforce to the community
Workforce outcomes	A culturally competent workforce; Seamless continua of care; Emphasis on patient centeredness; Accessing services earlier; Clear service pathways; Enhanced nurse leadership
Workforce issues	Prioritizing training needs; Models of care focussed on reducing loss of function and supporting continued community; Integrated teams supported by specialists; Support for provincial and rural services; Families acknowledged as principal carers; Funding focus on care planning not episodic care; Service designs are patient centric

### Results of scenario construction

Following our method, we took Table 4’s data to develop a normative scenario. Into this we blended the workforce actor issues to create alternatives. Two alternatives resulted, representing two diverse development paths, one based on the most divisive issues from the actor analysis, the other based on issues that created less but significant actor disagreement as controversial issues (Table 5). Each scenario was given a title to reflect its representative themes and principal issues (Table 6). The resultant scenarios are provided as an additional file at the end of the article.

**Table 5:** Actor data used to develop the alternative scenarios

Scenario	Divisive issues	Controversial issues	Potential actor behavior
Alternative 1	Costs and funding, New models of care	Leadership Shortages of medical workforce	Industry structure a barrier, Few incentives to change delivery, Scope of practice change, Funding flexibility may shift behavior but may not.
Alternative 2		Workforce profile Shortages of medical workforces Health workforce training Aging workforce Structure of health workforce New and extended roles Aging workforce Reliance on IMG & OQN	Access to training, Developing support networks for informal and family based care, Flexible service configurations based on regional and population needs Requires an overall strengthening of OPH workforce.

**Table 6:** Scenarios resulting from the construction procedures

Type	Name	Theme	Issues
Normative	Fit and Functional	A collective vision formed out of clinically led groups’ ideals for future Older Persons services and their delivery	Aging population, policy of integrating care, increasing use of patient centric and co-located care models
Alternative	Care’s Evolution	How sector resources are to be (re) distributed to facilitate community-based models of care	Commitment to quality, new roles, appropriate skills, staff attraction and retention and a network model of continua of care
Alternative	Transitioning Workforces	Who will do the work and where becomes more aligned with community expectations and needs	Diversity and the changing face of the workforce, valuing the caring role and attracting, training and retaining carers in extended roles

### Delphi panel progress

Even though our initial panel size was small, we made the decision to remove panellists who had made little or no contribution to the first two rounds as they would have contributed little to the initial data [72]. Question round response rates varied, reflecting participation rates and the effects of panel attrition. We collected commentaries or contacts with panellists to help us understand attrition causes, which indicated that participant time poverty was the main issue.

The panel closed at round four, as through attrition we had reached the minimum recommended panel size (five participants). Nevertheless, by this stage more than half of the scenario and policy statement items had reached response stability.

### Results of scenario evaluation

We evaluated our scenarios using the three questions. Of the four items affected by corrupt data, two displayed trends towards the stability threshold, while the other two provided no conclusive probability results (Table 7). This shows that the panel found all of the scenarios to be largely desirable and valid, with one of the alternatives, Care’s Evolution, being seen to be more desirable than the normative, Fit and Functional.

**Table 7:** Scenario ratings

Scenario	Rating		
	Desirability	Probability	Validity
Fit and Functional	Desirable	Either way (trending)	Reliable
Care’s Evolution	Very Desirable	No Result	Reliable
Transitioning Workforces	Desirable	No Result	Reliable (trending)

To understand these results’ contexts we further analysed the stable items to determine whether consensus or contention was experienced during the rating process. This analysis revealed that the panellists were in moderate consensus over their rating of the three scenarios and the options presented within them (see Table 8). For those items with contention results we reviewed the panellist comment data. These showed that for Fit and Functional’s validity, the panel was concerned about the sector’s leadership and stakeholder commitment to see this scenario through, while for the desirability of Transitioning Workforces, the scenario was seen as both aspirational and untested, with a review of the scenario’s assumptions suggested.

**Table 8:** Scenario stable item response characteristics

Scenario	Item	Characteristic
Fit and Functional	Desirability	Consensus
	Validity	Contention
Care’s Evolution	Desirability	Consensus
	Validity	Consensus
Transitioning Workforces	Desirability	Contention

In general, the panel found that “overall, the scenarios are valid” with the issues being “clearly articulated”, although the scenarios may not have been as “older people centric” as required. Even though there was some general agreement, the panellists’ did not universally endorse the scenarios as written. While Care’s Evolution was the most desirable, being favoured for its “broad health care focus including preventive care and dignity”, and its reliable premises, a panellist noted that “funding adjustment [is] necessary to follow patient needs and shift resources”. Commentaries from the inconclusive probability items for Care’s Evolution and Transitioning Workforces revealed that panellists’ had little confidence in the sector’s stakeholders to make decisions that corresponded with the scenarios’ visions by questioning how the actors would apply their power and influence and if the actors would just act in their own self-interest.

The panellists also commented that the scenarios did not go far enough, with doubts cast over whether these scenarios would meet the needs of populations whose care and wellbeing are not presently well served. Further, integrated care’s definitional variation also may have affected panellist interpretation, with some suggesting that more detail was required.

### Results of policy statement development

The inductive analysis of round one’s open-ended questions led to twenty-three policy statements being formed. These encompassed a range of policy issues about the transition from now to the future options concerning leadership, funding, models of care, transitions strategies, the role of the family, the increasing importance of community care workers and sector wages. The frequency of the referential codes for each policy statement, which ranged from fourteen to one, provide an indicator of how the panellists felt about the issue.

As this article is focussed on presenting our approach rather than results and content, we will provide a sample of the policy statement analysis to clarify our approach. Thus, in Table 9 we present four policy statements, selected by code frequency, as examples.

**Table 9:** Selected policy statements

#	Policy Statement	Code frequency
11	Wage rates in community and care based roles should be linked to funding increases and skill levels.	7
12	More good quality current workforce data should be gathered on choices, experiences, motivators and the sector’s attractors, to provide indicators for valuing and maintaining the workforce.	8
16	Care should be taken to ensure that the care workforce is not compromised through service transitions.	7
18	New service models should be the ‘continua of care’ type, centred on patients/whanau where the workforces have the appropriate skills at each point of care.	14

### Results of policy statement analysis

We evaluated the selected stable items for consensus or contention by reviewing the item-central tendency statistics, a visual inspection of histograms and a rereading the panel comment data on the ratings to understand panellist rationales (Table 10).

**Table 10:** Selected policy statement ratings and response characteristics

#	Item		Response characteristic
	Desirability	Feasibility	
11		Feasible	Contention
12	Very Desirable		Consensus
16	Very Desirable		Consensus
18			Not stable

We can see that the stable items 12 and 16 record consensus for their desirability, while item 11’s feasibility is contentious. This contention is derived from panellist differences over whether improved funding will translate into better sector wages. Item 18, the statement directly related to the preferred care model, had the highest frequency of references, but never-the-less it failed to reach stability. Reviewing the statement’s comments, we found that the panel saw this statement’s desirability being related to the existence of “integrated teams” and the presence of “good leadership”, with their feasibility doubts due to context dependency, service complexity and a reliance on inter-sectoral cooperation and capacity as patients move from and between various OPH and primary care services.

## Discussion

The aim of the article is to present and discuss scenarios and their analysis as a novel health workforce planning and policy analysis tool. We achieve this by following van der Heijen’s [34] option of using scenarios to identify and describe social and professional interactions that lead to an understanding of the processes that lead to a future. In doing this, we are not seeking perfect consensus on an idealised prospect. Rather, we are trying to explore opinions and identify positions that point us to issues that may impede or enhance the reaching of integration goals and, importantly, elicit signals for an understanding of the dynamics and the needs of its workforces. Thus, the data we derive informs us as to how stakeholders may interact and to identify what values and behaviours they would need to express for the described situation to be realised [49]. In a successful networked model of care we would expect to see stakeholders exhibiting mutual self-interest, cross-boundary connectedness and interprofessional support [92], though we found the panellists questioning how deeply these characteristics are held or expressed by the sector’s actors. This reveals an important aspect of scenario analysis; that it can act as a rehearsal of a policy’s implementation, with the resulting commentaries providing service and workforce planners a further means to identify the policy implications for service outcomes, its workforces and wider stakeholders.

Allowing for this insight is our choice of analysis method. As indicated previously, the policy Delphi allows for a situation or policy to be canvassed widely and the results and their interpretation are supported by techniques that reveal division and explore dissention and do not rely on consensus [55, 90]. This becomes important when delving into why certain scenarios are seen to be more attractive than others or why their validity comes into question. Thus, we can derive a better understanding of why a situation or policy statement is more favourable for some but not others and what cautions need to be exercised before a policy is presented or implemented. Indeed, we found that even for those items that the panellists’ deem important, contention and lack of response stability can occur. Taking his approach therefore complements the numbers-based workforce planning that drives most policy, particularly those to relieve shortages, by reducing the focus from the short-term and urgent and emphasising outcomes [40].

On many occasions workforce issues are not well reflected in health policy development [93] nor in a health system’s governance mechanisms [94]. One proposal to improve on this is to promote the commitment of all professionals and sectors in the policy development process [93]. Though often as not, patients are left out of policy or health planning, or, have little influence even when canvassed [60]. This would indicate that better engagement and involvement processes for patients and their families is required when attempting to integrate care and its service design and policy development processes.

However our approach is not without its detractions. We acknowledge the methodological limitations of Delphi, including participant time poverty and panel attrition [81, 95]. Even though we applied many of the Delphi method’s optimisation recommendations, we still experienced limiting issues. The decision to survey online [71, 87] was successful to some degree with reduced survey administration and data handling intervals. Though these did not necessarily improve participation or response times. The online option assumes that people have the requisite skills and introduces additional issues. Our experience suggests that another form of opinion polling or ranking method that also accounts for dissention could be chosen to refine the method and avoid some of the Delphi’s limitations. However, we must remember that any technique will still require a population of respondents, many of whom will be time poor.

There is also the limitation of the scenarios themselves. There is a range of methods to enable scenario creation [38]. Using another method may have produced scenarios that were more or less desirable, probable or valid, or have been more assertive in pushing boundaries. That said, scenarios need to be plausible and a more expansive approach may have produced a scenario too outlandish for the panel to accept and undermined the process [57].

Similarly, another limitation is the lack of a consistent definition for integrated care. Not having a common understanding of what is being discussed impedes statement formulation from panellist points of view. The scenarios we presented overcame this to some extent, by stating or implying what integration may look like, though some panellists still asked for more detail. However, developing scenarios with competing integrated care definitions could also be a way for policy makers and service planners to present their versions of integrated care by drafting narratives that reveal how patients and the workforce are proposed to interact and how a care model’s processes may operate differently from the present [59].

Lastly, there is the possible cultural barrier to adoption of our approach. Policy makers tend to be risk-averse and preferring certainty or predictability, hence the lack of widespread use of scenarios in policy making [45, 46]. Despite this, there is an emerging recognition that agencies should seek a diversity of workforce planning tools to overcome uncertainty [39, 96].

## Conclusion

In conclusion, we have presented an approach to address workforce planning uncertainty associated with integrated care. Our method goes past the traditional role of numbers-based delineation of workforce planning to identify issues and policy kernels for integrated care workforces. While our method is unique and has only been applied in New Zealand’s workforce planning environment, it shows potential for other health workforce policy and planning systems. Our approach provides an example of how policy makers and planners can use scenario analysis as a tool to identify workforce policies and to rehearse their implications, particularly when attempting to address integrating care. It also contributes to overcoming a significant weakness of health system governance by identifying health workforce-centric polices.

## Supplementary information

**Additional file 1.** OPH Scenario Set.

## Data Availability

Supporting data are available upon request from the corresponding author.

## Abbreviations

HWNZ: Health Workforce New Zealand
OPH: Older Persons Health
MM: Mixed Methods
