# Supplementary material for: Rethinking workforce planning for integrated care: using scenario analysis to facilitate policy development
Source: BMC Health Serv Res. 2020 May 15;20:429. doi: 10.1186/s12913-020-05304-4 (PMC7227104; doi:10.1186/s12913-020-05304-4)
Supplement: Supplementary file 1 — Additional file 1. OPH Scenario Set. [file 12913_2020_5304_MOESM1_ESM.docx]

**FIT AND FUNCTIONAL**

*Fit and functional* presents a collective vision formed out of clinically led groups’ ideals for future Older Persons services and their delivery.

The scenario is a compilation of the ideas expressed in Health Workforce New Zealand’s Work Service Reviews, which relate to service groups within the Older Persons Health sub sector.


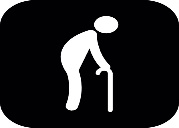

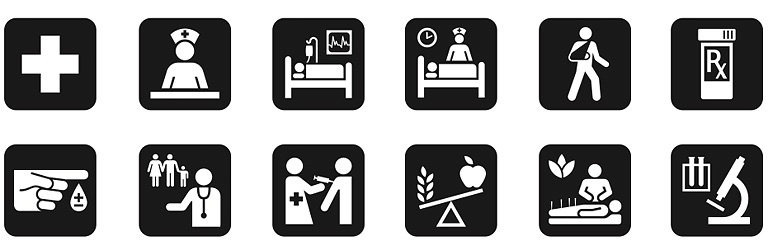

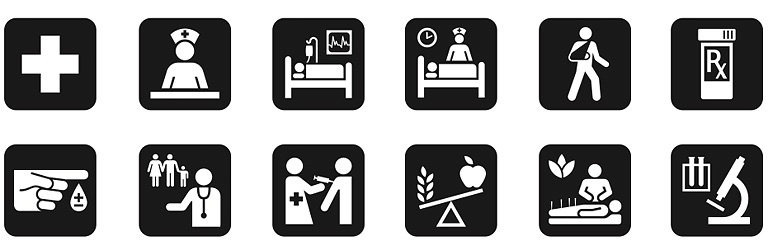

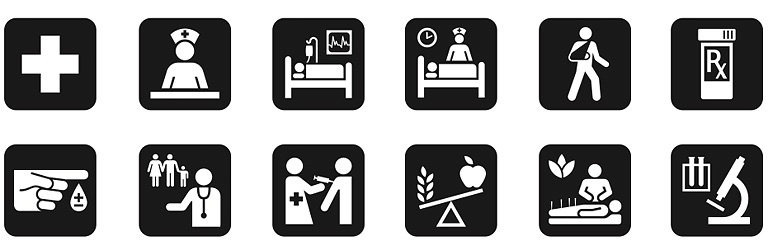

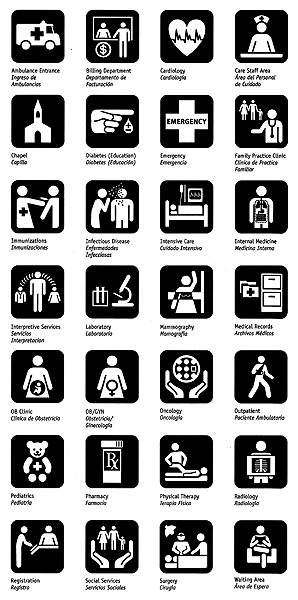

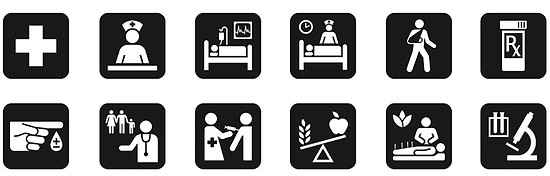


Older Persons Health sub sector Normative Scenario page 1

In response to demographic projections and expectations of a fitter and longer living older person, the health system has shifted to meet these new sets of demands. The Older Persons Health (OPH) landscape is now based on integrated care teams, supporting older people and their families to maintain and extend functionality preferably in their own homes. This is backed by significant investment in informal carers and the non-regulated workforce who are able to utilise information technologies that enable improved patient data collection and care planning.

Significant changes have occurred in the workforce with professionals shifting from institutional environments at one end of the spectrum, to a concerted effort to value the home based and informal caring workforce at the other. Enabling this was a stepwise approach to systemic funding and model of care adjustments to facilitate the transitions.

**Working across a continua of care with managed transitions**

The OPH workforce continues to work across a number of care environments and is more integrated and culturally competent, now working as part of multidisciplinary teams.

The teams deliver effective care, which is integrated along a seamless continua (residential, day facility, in-community and outreach services) with care planning and coordination enabling optimum health outcomes and independence for patients. Community based older persons care is strongly integrated with Primary healthcare (PHC), with provider arrangements spanning specialist, generalist, nursing, pharmacy and allied health domains.

**How did we get here?**

Models of care have changed, focussing more on reducing the loss of function and meeting the needs of the individual, thus changing the funding foci from episodic care to strengthening care assessment and patient journey planning. This includes a commitment to earlier specialist involvement and services that are convenient for the patient rather than provider.

Critical to achieving the change was acknowledging and valuing the role of the family as a primary support system. Gradually, informal and family carers have become empowered and are now routinely included in working with providers and specialists and have improved access to information about the best community based care and wellbeing options. Significantly, diversity in the workforce has been balanced, in terms of ethnicity and skills levels. There is improved access to culturally competent care, with specialist services centred where most people live.

Care and delivery leadership has been developed across the workforce supporting the move of specialist services from hospitals into the community to facilitate how the care is now delivered.

Workers from across the spectrum are involved in contributing to care and providing data through the ubiquitous use of technology. Rural and provincial service access, e.g. after hours GP’s, which previously presented potential access problems, has been maximised through the use of outreach and mobile options and introducing specialized nursing services working in partnership with PHC providers as part of the sub sector’s multidisciplinary nature.

Underpinning the change has been a commitment to strengthening access to and the content of training and continuing professional development, particularly in aspects of gerontology.

**Signposts**

- *Acknowledging the poor public perception and value of caring and older person’s healthcare.* The New Zealand Human Rights Commission’s report voiced many in the community’s concerns of the state and limited value placed on care of older people in New Zealand.

**Source**: New Zealand Human Rights Commission. (2012). Caring counts Tautiaki tika: Report of the Inquiry into the Aged Care Workforce.

- *Nurse Practitioners (NP) in OP care.* Changing model of care ensures advanced care plans can be delivered by nurse practitioners supported and supervised by specialists and practitioner teams, in part to offset regional shortages of GP services in communities and residential care settings.

**Source**: Peri, K., Boyd, M., Foster, S., & Stillwell, Y. (2013). Evaluation of the Nurse Practitioner in Aged Care.

- *Comprehensive Care Planning.* The Comprehensive Clinical Assessment Project is about establishing the use of a standardised assessment tool that uses software designed to improve the care of older people in residential care facilities.

**Source:** http://healthitboard.health.govt.nz/our-programmes/shared-health-information/common-clinical-information/comprehensive-clinical

- *Specialist roles in Primary Health Care*. To support community based models of aged care specialist PHC positions have been piloted and evaluated.

**Source:** King, A., Boyd, M., Carver, P., & Dagley, E. (2011). Evaluation of a gerontology nurse specialist in Primary Health Care: Case Finding, care coordination and service integration for At-Risk Older People

- *Commitment to improving roles and training.* The Kaiawhina Worker Workforce Action Plan 2014 has been developed and is responding to non-regulated workforce training needs and gaps.

**Source:** http://www.careerforce.org.nz/news/careerforce-news/kaiawhina-workforce-action-plan-gets-thumbs-up-at-the-careerforce-conference/

**Future Signals**

- *Continua of care options with continual high quality care.* The introduction of enhanced and easy access to training is available to workers across the continua of care along with focussed support and integration of informal and non-registered workforces.
- *Comprehensive system of care.* Service and facility design accounts for the increasing numbers of + 65s requiring short stay and transitional care. Care will be based on evidence with inter-disciplinary education enabling functional multidisciplinary teams using better use of technology to implement patient centred care models. Models of Care refer to the whole care continuum and explicitly define transitions and their management.
- *Integration for health outcomes.* The sector’s models of care refer to the whole continuum of care and explicitly define transitions and their management. The implementation of these required the development of new skill sets and attitudes surrounding how care is now delivered with a focussed shift from the previous institution or service siloes towards the individual's functioning and wellbeing.
- *Trained to perform.* Increased education and training based in gerontology improves the value of care workers and nurses and their career paths.

**Engagement Questions**

- Who should be leading the service transitions from the institutions into community settings?
- What changes to funding would support new models of care and provision?
- How will the commitment to develop the non-regulated workforces translate into increased attraction and pay?

Older Persons Health sub sector Normative Scenario page 2

**CARE’S EVOLUTION**

*Care’s evolution* recounts how sector resources are to be distributed to facilitate community based models of care. Resources are reconfigured to meet the pressure of rising demands, expectations and the devolution of older peoples’ health services into the community.


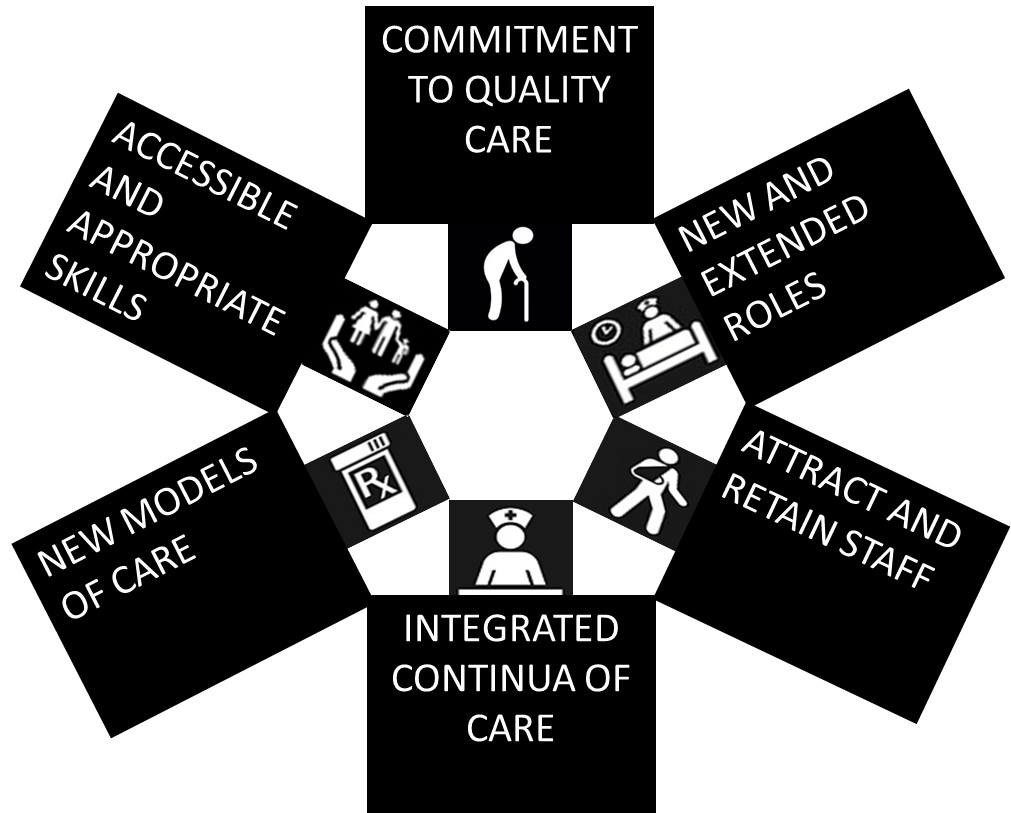


Older Persons Health sub sector Alternative Scenario 1 page 1

Faced with increasing pressure on system performance the government implements an investment approach for healthcare funding. These changes seek to attend to the formerly complex bundle of condition based or specialty funds, reinforced by silo arrangements and narrow models of care. Coupled with this is that the system is frozen through a combination of a perception of weak leadership and policy, with a fragmented industry whose actors are in perpetual rivalry over minor funding increases.

Key to the change approach was its encouragement of service mixes that improve system financial sustainability while improving outcomes for patients. Thus, service designs are now required to refer to their place in the patient journey and demonstrate integrated solutions, including links with social services.

Prompted by longer planning timeframes, increasing numbers of providers begin the process of change, resulting in workforce reorientation. Rather than taking short-run approaches to filling their gaps, providers can now invest and implement changes to work, value and skill levels. The funding timeframe also provides the added effect of limiting cost shifting and gaming behaviours. The funding system’s incentives are equitably distributed across the sector, with patients being the significant beneficiaries.

As people reside longer in their own home, patients enter residential care with more acute conditions. This requires higher levels of workforce skill to be provided in these settings, including improved hands-on care skills and emerging roles developed to address rising acuity. In more dense populations, private residential care has been able to remain viable, although in the regions providing residential aged care is still sometimes precarious.

To maintain an even coverage of care, the regional environments required additional attention. A mix of incentives and different care models were invested in. This develops knowledge, providing data on what works and the evidence for how to. This attention encouraged increasing diversity of service provision, including sharing across public-private ownership ensuring that optimum levels of care are feasibly possible. A range of mixes of public and private provision are regularly found, particularly in locations where scale advantages cannot occur due to declining populations and lack of investment.

Part of the provision of an appropriate care continua is acknowledging the role of informal care. Care models have responded, to where the family and its role as a principal care provider is now central. Determined by their care plans, some individuals are in positions to choose a range of aspects of their own care, with increasingly permanent relationships with their care givers and exercising more control over their lives. For others, traditional care options remain, with the care givers taking more part in care plan data collection and reporting.

In this team-based environment Nurse Practitioners are common place, particularly where local GP capacity is limited. There is increased involvement of pharmacy and allied health participation in individual care plans and to provide monitoring data. Critical to this evolution was the open confrontation of wage differentials between services, with solutions based on the importance of skills, competencies and values across the workforce continua.

**Signposts**

- *Funding directions encourage devolved patient focussed services.* Early reports of the New Zealand Health Strategy update’s Funding and Capability reviews have signalled patient centeredness and the employment of new models of care are priorities.

**Source:** Topham-Kindley, L. (2015, 27 July). Shake up of primary care funding signalled in leaked documents, NZ Doctor.

- *A range of funding mechanisms have been identified dependant on patient capacity and need complexity.* Home based support for older people has been identified an area where improvements can be made to how services are paid for and delivered.

**Source:** New Zealand Productivity Commission. (2015). More effective social services.

- *Increasing attention is being paid to the barriers and enablers of integrated care models and policy.* While integrated care is an attractive proposition it remains a new frontier. Increasingly research on the models and their policy contexts that make integrated care effective and deliver results to patients is now becoming available.

**Source:** International Journal Integrated Care 2015; Special Issue: Integrating Care to Older People and those with Complex Needs: http://www.ijic.org/index.php/ijic/issue/view/105

- *Workforces are determined by model of care redesign and funding mechanisms.* Future workforces are at risk of continuing to be driven by institutional rather than patient needs. Model of care design sets the parameters for the workforce, where the work will be undertaken and the skills required to carry it out.

**Source:** Imison, C., & Bohmer, R. (2013). NHS and social care workforce: meeting our needs now and in the future.

**Future Signals**

- *Care continua design improves service responsiveness.* Process design principles and techniques have been used when designing service continua so that patient flow, task allocation and the physical layouts of care facilities are improved, changing the number and types of staff required.
- *Align the workforce to need rather than by service provision.* The workforce is determined by the needs of the sector. Roles situated closer to patients grow in number as treatment and care in the community rises, while highly skilled specialists work in high needs residential care and dementia units. Community based care means that professional development has to include collaborative practice skills and inter-professional awareness and training.
- *Funding provides rewards equitably across the continua of care.* Benefits and incentives are shared across the workforce. This is particularly true for the increasingly important informal and family care roles. Informal carers needs are integrated into training and development activities. Volunteer groups are included in service planning and provision, not as an afterthought, but as part of the strong network of social care.
- *Transform how work is rewarded*. Remuneration of roles becomes more uniform primarily based on skills and levels of responsibility, which reduce the disparities between primary and secondary, public and private and medical and non-medical staff. Rewards and incentives engage workforces and attract people resulting in a more even distribution of people and skills.

**Engagement Questions**

- In this scenario, who loses?
- What would be the effects of these losses?
- What actions can actors take now to develop patient focused team based health care for older people?

Older Persons Health sub sector Alternative Scenario 1 page 2

**TRANSITIONING WORKFORCES**

In *Transitioning workforces,* a range of issues converge with respect to who will do the work and where. At stake is the ability for the sector to become more aligned with community expectations and needs by broadening diversity in skilled roles while strengthening and sustaining the quality of care.


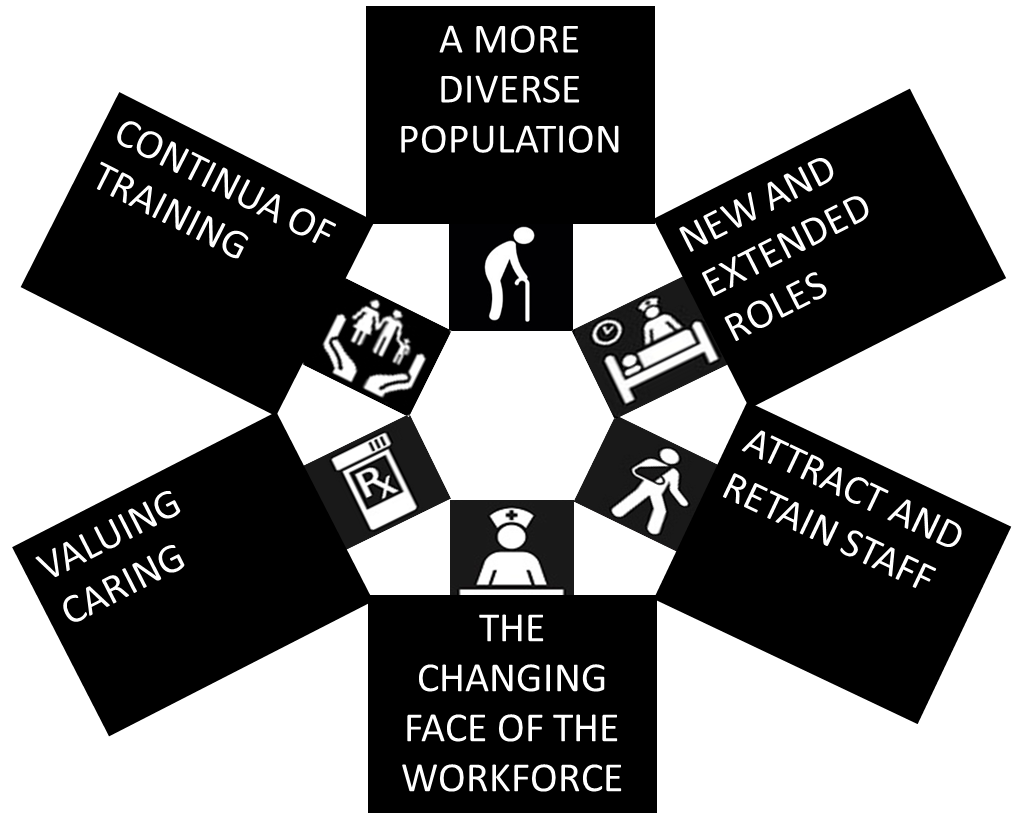


Older Persons Health sub sector Alternative Scenario 2 page 1

Community based models of care have placed increasing responsibility for services to be provided in older people’s homes and on the role of informal caring. As service devolution proceeds, significant effort is placed on strengthening and providing confidence in home and family care provision.

Workforce shortages have been a catalyst for change. Health providers are now proactively working with PHOs and DHBs to produce integrated models of care that match local needs. These models require a range of new and extended roles to ensure care is provided at the level required. In some cases due to low General Practitioner numbers or access difficulties, Nurse Practitioners (NP) are increasingly being used along with telemedicine options to maintain high quality community based care. A focus on interdisciplinary training has improved communication and care planning. Care options and pathways are now widely known and accessible. Patients and their families are frequently part of making decisions and choices, ensuring independence and functionality is maximised.

A focus on skills and accessible modularized training has led to better engagement with families who provide care and a better understanding of their needs and the skills and information that they require. Systematic feedback and input from carer networks provide important data on what training or information is needed and to what level. Some family carers are taking their informal skills into formal care, formally qualifying and pioneering this entry pathway to community based care work.

Equally, the access to and the facilitation of training finds institutional providers committing more and more to training and workforce development. The desirability of older persons care and treatment has been influenced by the strong focus of the Government, education institutions and sector representative groups who have jointly determined training pathways to access essential skills and their recognition.

The workforce is now mostly full-time, relieving shortage pressures within workplaces and contributing to the sector’s reduced reliance on overseas trained staff. Much of the care workforce is studying while they work, but this has only recently emerged following how the work is organized and distributed. As a result, learning has become more accessible and more frequent and in the workplace. This has lifted the workforce’s skill levels to meet the demands of rising acuity and multi-morbidity issues as the patient groups age.

As working in the sector has become more attractive, services are now better able to met the needs of the increasingly diverse New Zealand. Workforce ratios align closer to population. A broader range of roles available across the care continua have enabled more diverse and identifiable career pathways. This has seen increasing numbers of senior staff from Maori and Pacific peoples backgrounds, some working as NP aged care specialists, who are integral to continuing high quality community based care and its planning. This pattern is also reflected for specialist medical practitioners whose role supports generalist and community based practitioners, and maintain the strong values of patient focused care through their professional and clinical leadership.

**Signposts**

- *The characteristics, the skills and the competencies of the un-regulated workforce are becoming known.* A workforce profile on who makes up the un-regulated workforce and their skills has been produced.

**Source:** Twaddle, S., & Khan, M. (2014). Health and Disability Kaiawhina Worker Workforce, BERL.

- *Attention is being paid to the role of informal caring in New Zealand communities*. Data is emerging on the needs and value of informal caring in New Zealand.

**Source:** Grimmond, D. (2014). The economic value and impacts on informal care in New Zealand and The New Zealand Carers' Strategy Action Plan for 2014 to 2018 , https://www.msd.govt.nz/about-msd-and-our-work/work-programmes/policy-development/carers-strategy/ .

- *Limited diversity in the medical and nursing workforce is acknowledged.* Maori and Pacific peoples are under-represented with a closing gender gap in the medical workforce, a situation that is mirrored in the nursing workforce.

**Source:** Ministry of Health. (2014). Health of the Health Workforce 2013 - 2014: A report by Health Workforce New Zealand.

- *Aged care providers acknowledge that valuing staff is integral to a sustainable business.* Even though sector funding has not risen greatly some care businesses are substantially raising pay and improving access to training.

**Source:** Ground-breaking pay rise for Metlifecare staff. Radio New Zealand News Online. http://www.radionz.co.nz/news/national/282487/ground-breaking-pay-rise-for-metlifecare-staff

- *Telemedicine is becoming an option for the aged care environment.* Telemedicine, while not common, is entering New Zealand’s aged care sector as mobile device ownership improves.

**Source:** Denman, G. (2013). Telehealth in aged care. http://www.insitemagazine.co.nz/issues/july-2013/telehealth-in-aged-care/#.Vg7TDSu4Eq5.

**Future Signals**

- *Access to training and uptake is a core part of system capacity.* Education providers work closely with service providers and utilise older semi retired workers to improve access to and provide accessible training and professional development for the non-regulated and family care workforces.
- *The perception of the sector changes with work seen as valuable and professionally attractive.* Caring is more widely valued in the community. This value translates into increasing rates of pay, status and public appreciation. Professional roles are now more attractive as specialist positions become more integrated and team based and care roles are more involved in day-today aspects of care planning and patient monitoring.
- *Less reliance on IMG and OTN to fill gaps*. The practice of importing doctors and nurses to fill position in older persons services is reduced as new models of care replace the multiple silo model. Designing services in collaboration with patients means that effective services can be developed and trialled. As skills rise care and rehabilitation roles are better distributed through the workforce, situated closer to the patients.

**Engagement Questions**

- What are the catalysts that will shift the workforce to identify with a continua of care?
- How should work based training develop to involve and engage lower skilled workers?

Older Persons Health sub sector Alternative Scenario 2 page 2
